# Single-Cell Sequencing: Ariadne’s Thread in the Maze of Acute Myeloid Leukemia

**DOI:** 10.3390/diagnostics12040996

**Published:** 2022-04-15

**Authors:** Immacolata Redavid, Maria Rosa Conserva, Luisa Anelli, Antonella Zagaria, Giorgina Specchia, Pellegrino Musto, Francesco Albano

**Affiliations:** 1 Hematology Section, Department of Emergency and Organ Transplantation (D.E.T.O.), University of Bari ‘Aldo Moro’, 70124 Bari, Italy; immacolata.redavid@uniba.it (I.R.); maria.conserva1@uniba.it (M.R.C.); luisa.anelli@uniba.it (L.A.); antonella.zagaria@uniba.it (A.Z.); pellegrino.musto@uniba.it (P.M.); 2 School of Medicine, University of Bari ‘Aldo Moro’, 70124 Bari, Italy; specchiagiorgina@gmail.com

**Keywords:** single-cell DNA sequencing, single-cell RNA sequencing, acute myeloid leukemia, clonal heterogeneity, clonal evolution

## Abstract

Acute myeloid leukemia (AML) is a haematological neoplasm resulting from the accumulation of genetic and epigenetic alterations. Patients’ prognoses vary with AML genetic heterogeneity, which hampers successful treatments. Single-cell approaches have provided new insights of the clonal architecture of AML, revealing the mutational history from diagnosis, during treatment and to relapse. In this review, we imagine single-cell technologies as the Ariadne’s thread that will guide us out of the AML maze, provide a precise identikit of the leukemic cell at single-cell resolution and explore genomic, transcriptomic, epigenetic and proteomic levels.

## 1. Introduction

*“Tum Ariadna: <Ego vero tibi auxilium feram: ecce filum quod tibi viam monstrabit.>*

*Post monstri interfectionem, vir, filum tenens, exitum labyrinthi facile repperit”.*

*Thesei mythus*

*“Therefore, Ariadne said: < I will help you. This is the thread that will guide you.>*

*The man killed the monster and overcame easily the maze thanks to the thread”.*

*The myth of Theseus*

In this famous myth, Theseus is able to solve the maze by rewinding Ariadne’s thread; in the same way, single-cell technologies may disclose the acute myeloid leukemia (AML) labyrinthine complexity. AML study has been focused on identifying genetically heterogeneous neoplastic cell populations for several decades. From cancer initiation to diagnosis and progression, leukemic cells undergo clonal evolution, acquiring several genetic and epigenetic alterations. Despite the enormous progress in understanding the leukemia pathophysiology, the disease is still highly challenging. AML is currently defined as an aggressive neoplasm characterized by different subclones capable of deeply impacting tumor evolution and the acquisition of therapeutic resistance, which are relatively rare and sometimes undetectable by traditional methods. The advent of next-generation sequencing (NGS) techniques has dramatically revolutionized the research and diagnostics of malignant diseases and greatly enabled an improved knowledge of cancer biology, including clonal evolution, transformation, adaptative selection and treatment resistance of leukemic cells [1]. In the AML field, NGS has allowed us to pinpoint numerous preleukemic mutations in the hematopoietic stem and progenitor cells (HSPCs) compartment, which drive clonal evolution and survival despite standard induction chemotherapy, leading to disease relapse [2]. Undoubtedly, bulk tumor cell analysis has allowed significant advances in cell populations and cancer treatment characterization. Nevertheless, standard bulk population sequencing is frequently unable to identify rare alleles, or unequivocally determine whether mutations co-occur within the same cell, so that a single-cell resolution may be decisive. The purpose of this review is to highlight how crucial the single-cell approach could be in the context of AML. Its capacity to evaluate the cell-by-cell potential of leukemic cells for proliferation, self-renewal and treatment resistance and the identification of leukemic cell populations with a “druggable” mutation, may help to guide the way out of the leukemic labyrinth.

## 2. Single-Cell Approaches in AML: A Future Outlook

Single-cell analyses can dissect intra-tumor genetic and epigenetic heterogeneity at single-cell resolution, leading to the identification of clones that accumulate that accumulate chemo/immunotherapy resistance factors, modulating prognosis and therapeutic response [3]. Accordingly, AML is characterized by an enormous molecular heterogeneity and the application of single-cell technologies could provide powerful insight into leukemia initiation, evolution and relapse [4]. Single-cell techniques have provided complete information about the genetic landscape, sub-clonal architecture, regulatory network, gene expression and proteomic profile of several malignancies [5]. Undoubtedly, in the AML scenario, the amount of biological information derived from a unique single-cell sequencing experiment far exceeds the yield of other more commonly used single-cell methods commonly used for investigating blood cancers, such as karyotyping, in situ hybridization, immunophenotyping, flow cytometry and mass cytometry [4]. Accordingly, dissecting cellular heterogeneity is a core single-cell DNA/RNA sequencing application. It assesses similarities and differences in genomic and transcriptomic profiles among different cell subpopulations that are undetectable by bulk DNA and RNA sequencing

## 3. Technological Panorama of Single-Cell DNA Sequencing (scDNA-seq)

Acquiring high-quality scDNA-seq data poses four fundamental technical challenges: adequate physical single-cell isolation; genome amplification of the isolated cell to obtain sufficient material for downstream analyses; appropriate querying of the genome to identify the variant under investigation; and data interpreting within the context of biases and errors that may be introduced during the first three steps [6]. Several methods can be employed to isolate a single cell, including mouth pipetting, micromanipulation, flow-assisted cell sorting, laser-capture-microdissection, serial dilution and microfluidics [7]. Each approach mentioned features a different accuracy, throughput, reproducibility and facility of use [8,9]. Before sequencing, DNA needs to be amplified by whole-genome amplification (WGA). Several WGA approaches have been described, including degenerate oligonucleotide-primed PCR [10,11], multiple displacement amplification [12,13], multiple annealing and looping-based amplification cycles and PicoPLEX [14,15]. It has been demonstrated that using a microfluidic device for the single-cell WGA is associated with a decrease in contamination [16]. Recently, an innovative two-step microfluidic droplet procedure has been developed that ensures efficient and massively parallel single-cell PCR-based barcoding. It consists of the encapsulation of individual cells in droplets, the lyses and the lysate’s digestion with proteases before genomic DNA amplification. After the protease’s inactivation, droplets containing the genomes of individual cells are barcoded and amplified [17]. The type of genomic interrogation, which may be whole-genome sequencing, target sequencing or whole-exome sequencing, needs to be assessed according to the aim of the study [18]. Using these types of data, it is possible to trace the mutational history of driver genes. Recently, a single-cell study focused on myelodysplastic syndrome (MDS) patients in progression to secondary AML (sAML), revealing a crucial role of a stem cell cluster that was not individuated in the MDS phase but became predominant during AML progression. Surely these results display a nonlinear progression to sAML and have implications on current oncology approaches [19]. Several data may be collected through the use of these single-cell approaches. It has been shown that by interrogating the genotype and immunophenotype of single leukemic cells, it is possible to define the proteogenomic patterns of AML patients [20].

## 4. Clonal Evolution and Genetic Heterogeneity in AML

ScDNA-seq technologies offer a novel opportunity to better investigate cell types such as cancer stem cells, clarify the processes involved in cell fate transition and explore clonal complexity, previously less appreciated in bulk NGS. These new approaches allow accurate study of the AML clonal architecture at single-cell resolution. ScDNA mutation study has disclosed two main types of clonal evolution in AML, found in disease initiation and during progression or response to treatment or relapse: linear, in which new clones arise as a result of the acquisition of sequential new mutations, and branched, in which new clones derive from one parental clone, acquiring different mutations and maintaining different and parallel evolution processes [21]. Furthermore, scDNA-seq technologies underscore that each AML case constitutes a combination of distinct clonal populations, resulting in deep intertumoral and intratumoral variety [22,23,24,25,26,27]. In particular, it was found that clonal complexity increases from clonal hematopoiesis or myeloproliferative neoplasms to AML and continues to progress, since AML clones tend to acquire mutations, especially in signaling effectors and frequently occur in subclones populations [23]. Furthermore, the mutational landscape is complicated by the different contributions of several mutation combinations. Standard bulk population sequencing is often limited in determining the co-presence of mutations in the same cell. By contrast, scDNA-seq is a powerful strategy; in fact, several studies have demonstrated that combinations including *NPM1c* + *FLT3-*ITD or *DNMT3A* + *IDH2* are often associated with clonal dominance, whereas others such as *NPM1c* + *RAS* do not promote clonal expansion [23]. However, mutations associations seem to be central to AML progressions; at least 85% of AML cases present two or more mutations [28]. Several studies have revealed that most of these cases exhibit co-incident mutations in epigenetic modifiers, including *DNMT3A*, *TET2*, *ASXL1* and/or *IDH1/2*. Moreover, the co-presence of these mutations is found in the dominant clone in at least 80% of cases [23,29], underlining the crucial role of the altered epigenetic factor in increasing the advantage of the clonal subtype. By contrast, mutations in signaling factors seem to be mutually exclusive since scDNA studies revealed the presence of alterations in *KRAS*, *NRAS*, *KIT* and *FLT3* in different clones [22,23,29,30], co-occurring only in a few cases but not in the dominant clone [23]. These data suggest that their functional redundancy is not required for survival advantage. Morita et al. increased our knowledge of the AML clonal architecture, by investigating the largest group of AML patients at single-cell resolution. Single-cell studies have identified mutations more frequently found in the dominant clone, including *NPM1* (90%) and *IDH1/2* (75%), whereas *FLT3*, *NRAS* and *KIT* are less frequently observed (25%) [22,23,31]. Most mutations revealed by scDNA-seq were heterozygous, the most frequently mutated being *ASXL1*, *FLT3-non-ITD*, *DNMT3A*, *EZH2*, *IDH1/2*, *KIT*, *KRAS*, *NRAS*, *PTPN11*, *SF3B1*, *NPM1*, *TP53*, *U2AF1* and *WT1*. *JAK2* and *GATA2* gene mutations were often homozygous, whereas *NPM1c*, *FLT3-ITD*, *RUNX*, and *SRFS2* were heterozygous in some cases and homozygous in a minority of clones [22]. Another interesting finding is that a certain order of mutation acquiring is respected. Epigenetic modifiers tend to gain mutations earlier in the founding clone, while mutations in *NPM1*, *FLT3* and *RAS* tend to be acquired later [22,23,29,31,32], with some exceptions (such as *NPM1c* and *TET2*, which may occur early or later during clonal evolution) [22,31]. Moreover, it has been seen that even uncorrelated mutations affecting the same gene in AML subclones derived by branching evolution, maintain this order. These data suggest that clonal evolution and the order of mutation acquisition may be crucial for the AML pathogenesis and transformation. Furthermore, a few studies based on a combination of scDNA-seq and immunophenotype have demonstrated that AML complexity is increased by the correlation between clonal immunophenotypes and mutational acquisitions [23,33]. ScDNA-seq studies have stressed the extreme complexity of clonal diversity in AML, showing a high degree of heterogeneity, especially in rare cases harboring more than 30 different clones and up to 7 different gene mutations [23]. However, in most AML patients, 3–13 clones and 3–7 gene mutations are found [22,23,33,34,35], whereas 1–2 clones are generally the dominant ones [22,23]. Moreover, the affluence of each clone seems often to increase with the acquisition of a new mutation. By contrast, the abundance of the dominant clone appears to be inversely related to the number of subclones [23]. Normally, scDNA-seq is efficient as a means of disclosing the AML architecture and individuating the dominant clone. Still, in some cases, subclones are present in equal manner quantities [22,23], suggesting that subclones may cooperate with the dominant one during AML evolution. Moreover, Wu et al. have deduced an AML progenitor cell cluster, integrating single-cell analysis of approximately 190,000 cells [36]. They found that AML progenitor cells and HSPCs had several upregulated genes in common with the myeloid cells, particularly the ribosomal protein (RP) genes implicated in the p53 pathway. Despite the presence of common characteristics, AML progenitor cells differ among patients: those with RP upregulated progenitor cells had a poor prognosis. Previous studies have proposed the involvement of RP in tumorigenesis, demonstrating alterations in several malignancies [37]. In fact, they seem to provide advantages to neoplastic cells [38], perhaps through extra ribosomal functions, including proliferation, DNA repair and apoptosis, allowing the acquisition and preservation of a cancer stem cell phenotype [39,40]. Further studies are needed to clarify the role of RP genes in AML. Overall, these findings suggest that AML is characterized by a complex scenario of clones in constant evolution, and a clonal architecture influenced by different mutational implications, all aspects well disclosed by scDNA-seq.

## 5. Clonal Changes in Response to Treatment

Intratumoral AML heterogeneity is strictly related to therapy response, so many studies have focused on disclosing why patients may be refractory to induction chemotherapy or relapse after remission. Previously, bulk sequencing studies suggested that in these patients relapse may be related to the presence of a higher number of mutations at diagnosis, when compared to patients who have a longer relapse-free survival [32]. scDNA-seq can be used for comparing clonal architecture at diagnosis, remission and relapse, allowing resistant clones leading to relapse to be individuated and used as predictive markers of relapse after treatments [31]. To date, the AML patients’ range studied with scDNA-seq is still limited; it has been proposed that AML relapse may be subordinated to clonal architecture and defined by the combination of particular gene mutations. This hypothesis paves the way to determining the eventual prognostic value and role in therapeutic choice [41]. In fact, it has been possible to discriminate minimal residual disease (MRD) clones involved in relapse and individuate patterns in clonal evolution that may confer a predisposition to relapse. In particular, it has been observed that a clonal heterogeneity decrease during remission was associated with a more prolonged relapse-free survival. This finding suggests that a clonal diversity increase may predispose one to relapse, underlying the fact that the clone type distribution and variety has more influence than the amount of mutations. Therefore, the co-occurrence of mutations in the same clone seems to confer a worse prognosis than the same mutations in different clones [31]. These investigations undoubtedly rely on a single-cell approach, thanks to its fine resolution. The possibility to detect as few as three mutation-harboring subclones may upgrade the current MRD monitoring strategies, given the prognostic value of specific clones with co-occurring mutations [17]. Single-cell strategies also provide a way to untangle the complex pathogenesis of relapses after allogeneic transplantation. In fact, a deeper investigation of chimerism allows an improved quantification and detection of different clones harboring single or multiple mutations, which is not possible or reliable using the classical bulk NGS approach. Moreover, a subclone can be discerned as pivotal for relapse, characterized by a precedent mutation rather than a de novo one [42]. Single-cell studies have improved our knowledge about the mechanisms of therapeutic action and resistance closely linked to intratumoral heterogeneity [43], especially regarding *FLT3* inhibitors used in relapsed refractory AML. Indeed, while bulk NGS studies described the insurgence of new mutations in the RAS pathway in a cohort of patients treated with *FLT3* inhibitor and the permanence of *FLT3* mutant clones, scDNA-seq revealed leukemic cells with the co-occurrence of these mutations [44]. Single-cell analysis has also suggested that a potential mechanism of therapeutic resistance may be transcriptional plasticity, by which leukemic clones can readapt [45], suggesting the importance of implementing epigenetic therapies [46]. The single-cell technique capacity to trace the clonal evolution from diagnosis through treatment at single-cell resolution offers an exclusive chance to investigate and define the clinical and biologic impact of AML clonal architecture and genetic heterogeneity, especially in terms of therapeutic strategies from a targeted therapy perspective.

## 6. Technological Panorama of Single-Cell RNA Sequencing (scRNA-seq)

The first scRNA-seq experiment dates back to 2009 [47]; meanwhile, technological progress has led to several commercial scRNA-seq platforms. The fundamental steps of scRNA-seq encompass single-cell isolation, the capture of RNA molecules, reverse transcription, cDNA amplification, library preparation, sequencing and data analysis. The platforms most commonly used to study hematological diseases have been reviewed by Zhu et al. [48]. Each of them lays claim to a different automated single-cell capture process based on the microfluidic chip [49], microwell array [50] or microdroplet system [51,52]. Alternative non-commercial platforms are achievable, including massively parallel RNA single-cell sequencing (MARS-seq) and SMART-seq3 [53,54]. Additionally, a new cellular method has been described, one that indexes transcriptomes and epitopes by sequencing, which can be easily integrated into the existing scRNA-seq platforms, allowing the coupling of cell surface protein expression and single-cell transcriptome information [55]. Although other scRNA-seq platforms exhibit differences in throughput, sensitivity, precision, cost and convenience, they represent a powerful approach to answering different biological problems. In light of the progress in our knowledge of the genomic scenario of hematological malignancies and immune landmarks, scRNA-seq can be exploited for AML surveillance, precise prediction of early progression and therapeutic management. In the AML context, several scRNA-seq applications have been reported, including the tracking of lineage and developmental relationships in heterogeneous but related cellular states [56,57,58,59]. Undoubtedly, one significant application of scRNA-seq is in identifying single-cell transcriptome clusters. Specifically, the study of gene co-expression patterns may identify co-regulated gene modules and assess gene-regulatory networks that are key to the definition of functional heterogeneity and cell type [60]. By comparing the transcriptional profiling of normal HSPCs to leukemic stem cells (LSCs), a genes subset that was LSCs-specific has been discovered that included both CD69 and CD36, enabling the use of these markers for identifying LSCs subsets with a variable self-renewal potential [61]. Furthermore, by combining high-throughput scRNA-seq with single-cell genotyping of recurrently mutated AML genes, it has been observed that monocyte-like AML cells also contribute to AML biology. These experiments have provided insight into the aberrant regulatory programs of primitive AML cells. They have identified differentiated malignant cells with immunosuppressive properties, contributing to altered T cell phenotypes and an immunosuppressive AML microenvironment [30,62]. Multipotent mesenchymal stem/stromal cells are crucial in maintaining and regulating stem cell function with cellular interactions and secreted factors within the niche [63]. The characterization of the entire stem cell niche has unveiled the pivotal role of the tumor microenvironment in disease progression [64]. Accordingly, it has been corroborated that developing AML leads to an altered mesenchymal osteogenic differentiation and decreases the regulatory molecules necessary for normal hematopoiesis; consequently, tissue stroma offers disadvantages for normal cells and enables the onset of leukemia [65]. In most studies, scRNA-seq was combined with other experiments to uncover AML pathogenic mechanisms, such as examining potential links between epigenetic and transcription heterogeneity [66]. Aging human HSCs increase malignant transformation risk associated with epigenetic deregulation. Investigating the epigenetics role in the AML pathogenesis, scRNA-seq analysis showed that the epigenetic changes program resulted from a true epigenetic reprogramming rather than the spread of a pre-existing leukemic subclone [67]. Furthermore, scRNA-seq is useful to study the alternative polyadenylation dynamics that are important for regulating gene expression, mRNA stability and efficient translation. Their involvement in cancer pathogenesis and development have been previously described [68]. Reportedly, alternative polyadenylation dynamics in AML patients were markedly abundant in pathways involved in leukemia development, suggesting that they may have a significant role in the AML pathogenesis [69]. These findings indicate that the implementation of scRNA-seq may ultimately contribute to the definition of a single leukemic cell identikit.

## 7. Conclusions

**Figure 1:**
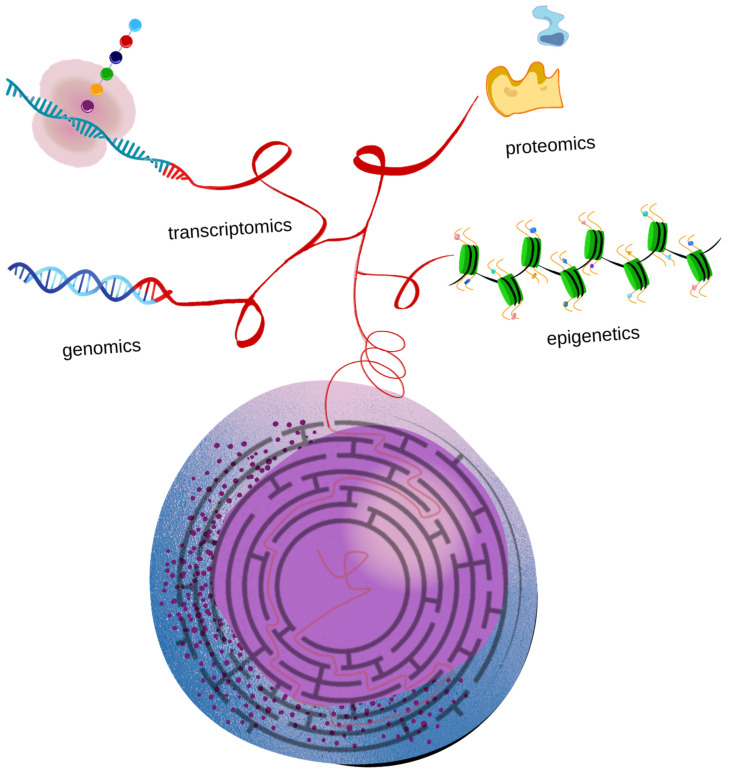
Single-cell studies of different biological levels allow for solving the complex AML maze.

Single-cell technologies are revolutionizing the knowledge of AML biology (Table 1), offering an unequalled chance to disclose the intratumor heterogeneity, identify rare cell populations and track clonal evolution. In our opinion, revealing the peculiar characteristics of the single cell, including proliferation potential, self-renewal and mechanisms of resistance, may be helpful to improve the identification of the malignant cluster to target. Moreover, these approaches can be exploited to better understand the molecular mechanisms underlying drug resistance and relapse in AML. It may be envisaged that in the near future it could be possible to make a complete scan of the single leukemic cell, gaining genomic, transcriptomic, epigenetic and proteomic information. Furthermore, single-cell technologies may be exploited for leukemic cells and the tumoral microenvironment, given their well-known role in leukemic support. The standard treatment strategies may be revolutionized, reinforcing the current individualized therapy approaches and improving patients’ prognoses. Considering all the potentialities reviewed, we believe that these technologies make the future look not so distant, since an accurate single malignant cell identikit from genotype to phenotype could encourage timely and targeted intervention in AML patients. Indeed, it will not be easy to introduce into routine clinical practice the accurate study of the several clusters of cells that make up and support the tumour growth, but even the maze seemed impossible to solve. Nevertheless, Ariadne’s intuition and Theseus’ perseverance were enough. In conclusion, single-cell technologies may constitute the escape route from the complexity of AML, just as Ariadne’s thread uncoiling and recoiling brought Theseus safely out of the Minotaur’s labyrinth (Figure 1).

## Figures and Tables

**Table 1:** Overview of single-cell sequencing studies in AML.

Single-Cell Approach	Aim of the Study	Object of the Study	Results	Ref.
scDNA-seq (cell sorter + Illumina)	Clonal heterogeneity	6 AML patients	Identified preleukemic mutations in HSCs	[26]
scDNA-seq (cell sorter + Illumina)	Clonal heterogeneity	3 MDS patients who progressed to sAML	Confirmed the clonal evolution and architecture of sAML originally detected by bulk methods	[24]
scDNA-seq (cell sorter + Sanger Sequencing)	Clonal heterogeneity	AML cell line Kasumi-1 and 1 inv(16) positive AML with germline CBL mutation	Characterized clonal composition and evolution of inv(16) AML (CBL) revealed the co-occurrence of several mutations in the same AML clone	[27]
scDNA-seq (cell sorter+ pyrosequencing)	Clonal heterogeneity *FLT3*-ITD primary AML	Patients enrolled on clinical trials of quizartinib in relapsed or refractory AML	Identified several cells subpopulation which underlies AML resistance to quizartinib	[43]
scDNA-seq (Tapestri Platform)	Clonal heterogeneity	2 AML patients at different key time points (~16,000 cells)	Identified cells harboring pathogenic mutations and uncovered complex clonal evolution within AML tumors that was not observable with bulk sequencing.	[17]
scDNA-seq (Fluidigm platform)	Clonal heterogeneity	10 cases of *NPM1* mutant AML	A preferential order of mutation accrual and parallel evolution of AML sub-clones was demonstrated.	[29]
scDNA-seq (cell sorter + Illumina)	Analyses of stem cell populations	7 MDS patients who progressed to sAML	The crucial role of diverse stem cell compartments is identified during MDS progression to AML.	[19]
scDNA-seq (Tapestri Platform)	Clonal architecture and clonal evolution of AML	2 AML patients at different key time points (2045 to 8619 cells/sample)	A precise picture of bone marrow engraftment and mutational profile of tumor cells from one assay was simultaneously characterized.	[42]
scDNA-seq (Tapestri Platform)	Resistance mechanism	3 AML patients at different key time points (4000–16,000 cells/sample)	Identified several patterns of clonal selection and evolution in response to *FLT3* inhibition	[44]
scDNA-seq (Tapestri Platform)	Clonal dynamics of AML from diagnosis to remission to relapse	14 patients with AML at different key time points (310,737 cells)	Discovered complex patterns of clonal heterogeneity and evolution that may predispose patients to relapse	[31]
scDNA-seq + protein-seq (Tapestri Platform)	Genetic and phenotypic heterogeneity	123 AML patients at different key time points (735,483 cells)	The mutational history of driver genes and observation of linear and branching clonal evolution patterns in AML was analyzed.	[22]
scDNA-seq + protein-seq (Tapestri Platform)	Clonal heterogeneity	123 AML patients (740,529 cells)	The complex ecosystem of clones that contributes to the pathogenesis of myeloid transformation has been identified.	[23]
scDNA-seq + Abseq (Tapestri + Abseq Platform)	Clonal heterogeneity	3 AML patients at different key time points (54,717 cells)	The study showed complex genotype-phenotype dynamics underlying the disease process.	[20]
scRNA-seq (Fluidigm C1 platform)	Transcriptional heterogeneity	Murine leukemia model	*DNMT3A*^R878H/WT^ mice-developed AML enriched in LSCs	[59]
scRNA-seq (Seq-Well Platform)	Transcriptional heterogeneity	16 AML patients (38,410 cells)	Identified aberrant regulatory programs of primitive AML cells and differentiated AML cells with immunosuppressive properties	[30]
scRNA-seq (10X Genomics platform)	Relationship between expression heterogeneity and sub-clonal architecture in AML	4 AML and 1 sAML patients (10,000–15,000 cells/sample)	Detection of expression heterogeneity in the absence of detectable genetic heterogeneity	[35]
scRNA-seq (10X Genomics platform)	Investigation of dynamic alternative polyadenylation involved in the mediation of AML	2 AML patients at different key time points (16,843 cells)	Extensive involvement of alternative polyadenylation regulation in leukemia development	[69]
scRNA-seq (10X Genomics platform)	Characterization of bone marrow stroma subpopulation	Murine leukemia model	Identified seventeen stromal subsets expressing distinct hematopoietic regulatory genes	[65]
scRNA-seq (Microwell-seq)	Clonal heterogeneity	40 AML patients (191,727 cells)	Identified a key AML progenitor cell cluster	[36]
scRNA-seq (10X Genomics platform)	Clonal heterogeneity	t(8;21) AML patients at different key time points (83,021 cells)	The heterogeneous malignant cells have unique characteristics that may evolve during disease progression.	[58]
scRNA-seq (Fluidigm C1 platform)	Molecular characterization of LSCs	AML samples with >50% bone marrow blasts and murine leukemia model	Established two distinct transcriptional foundations of self-renewal and proliferation in LSCs	[61]
